# Left atrial shunting devices: why, what, how, and… when?

**DOI:** 10.1007/s10741-025-10485-3

**Published:** 2025-01-25

**Authors:** Leila Anna De Lorenzo, Claudia Baratto, Davide Sala, Giovanni Battista Perego, Sergio Caravita

**Affiliations:** 1 Università Vita E Salute San Raffaele, Milan, Italy; 2 Department of Cardiology, San Luca Hospital, IRCCS Istituto Auxologico Italiano, Milan, Italy; 3 Department of Management, Information and Production Engineering, University of Bergamo, Edificio C, Via Pasubio, 24044 Dalmine, Bergamo Italy; 4 Dyspnea and Pulmonary Hypertension Center, Ospedale San Luca, Piazzale Brescia 20, 20149 Milano, Italy

**Keywords:** Heart failure, Interatrial shunt device, Left atrium, Pulmonary hypertension, Diastolic dysfunction

## Abstract

Left atrial (LA) hypertension is central in the pathophysiology of heart failure (HF) in general and of HF with preserved ejection fraction (HFpEF) in particular. Despite approved treatments, a number of HF patients continue experiencing disabling symptoms due to LA hypertension, causing pulmonary congestion, pulmonary hypertension, and right heart dysfunction, at rest and/or during exercise. LA decompression therapies, i.e., left atrial shunting through a specifically designed device (either implant-based or implant-free), are being studied in various forms of HF to alleviate LA hypertension and patients’ symptoms. Despite a solid background and favorable signals from initial non-randomized clinical trials, the quest for the optimal HF candidate for interatrial shunt devices is still an area of active research that at the same time is helping to better elucidate the intricate pathophysiology of HF(pEF).

## Why: rationale for the creation of a left atrial shunt in patients with heart failure

Heart failure (HF) is a clinical syndrome consisting of symptoms and signs due to cardiac structural or functional abnormalities. It is commonly classified according to the left ventricular (LV) ejection fraction (EF): HF with reduced EF (HFrEF, with EF < 40%), HF with mildly reduced EF (HFmrEF, EF 41–49%), and HF with preserved EF (HFpEF, EF > 50%) [1]. However, and irrespectively of the EF phenotype, high left heart filling pressure is a common finding in HF, either at rest or during exercise [2, 3], and it is associated with exertional symptoms [4], disease severity [5], pulmonary congestion [6], pulmonary hypertension, and right heart dysfunction [7–9].

LV (diastolic) dysfunction is thought to be the *primum movens* of high left heart filling pressure, through a variety of mechanisms including LV scarring, fibrosis, hypertrophy, and myocyte dysfunction [10]. However, the left atrium (LA) is not a passive bystander in HF pathophysiology: LA scarring/fibrosis and LA myopathy, or LA overdistension may contribute to reducing LA compliance and elevate LA pressure (or pulmonary artery wedge pressure, PAWP, as a surrogate for LA pressure) well above LV end-diastolic pressure [11]. Approved treatments for HF can improve patients’ hemodynamics, symptoms, and prognosis [1]. However, codified treatment exists mainly for HFrEF patients rather than for HFpEF or HFmrEF patients. Additionally, despite optimized treatment, HF patients may still experience disease progression and a high symptom burden, negatively impacting the quality of life.

These are among the reasons that led to elaborate LA decompression therapies, consisting of the creation of a shunt from a stiff LA to the more compliant right atrium (RA) or to the coronary sinus, in order to alleviate the LA pressure overload and its clinical consequences.

LA shunting is supported by a number of “incidental” clinical observations, including.

- Patients with severe mitral stenosis and atrial defect (Lutembacher’s syndrome) are less symptomatic than patients with mitral stenosis and intact atrial septum [12].
- Patients with LV diastolic dysfunction, like elderly people, can develop acute pulmonary edema after the closure of an atrial septal defect, since the elevation of filling pressure is no longer relieved by the atrial septal defect [12, 13].

Mathematical modeling has been subsequently applied to this background, in order to simulate the effects of the creation of an interatrial shunt on resting and exercise hemodynamics of HFpEF patients. These simulations showed that an interatrial shunt of about 8 mm diameter would reduce atrial pressure overload both at rest and during exercise in patients with HFpEF, all without compromising systemic cardiac output and without leading to an excessive increase in pulmonary vascular flow [14]. Indeed, too large a left-to-right shunt could result in right heart volume overload and potential right ventricular dysfunction.

## What and how: left atrial shunt devices and related clinical trials

Different types of devices have been developed. They are divided into two groups depending on an implant-based or implant-free approach. Each device has been tested (or is being tested) in dedicated studies, as detailed below. Notably, available evidence published so far mainly comes from cohorts of HFpEF and HFmrEF patients. Eligibility criteria for clinical trial entry, outcomes, and hemodynamic changes in the principal studies are reported in Table 1.

**Table 1:** Eligibility criteria and outcomes for left atrial shunting devices

	**REDUCE LAP-HF **[16]	**REDUCE LAP-HF I **[19]	**REDUCE LAP-HF II **[20]	**Rodés-Cabau et al. **[25]	**RELIEVE-HF trial **[27]	**ALLEVIATE-HF I e II **[32]	**AFR-PRELIEVE **[28]	**ALT-FLOW **[30]
Device	IASD (Corvia)	IASD (Corvia)	IASD (Corvia)	V-wave Interatrial shunt (first generation)	Ventura (V-wave)	ALV-Sistem (Alleviant)	AFR-Device (Occlutech)	APTURE (Edwards)
Number of treated patients (device vs. Sham procedure)	64	44 (22 vs. 22)	626 (314 vs. 312)	38	508 (250 vs. 258)	28	36	69
Main eligibility criteria								
NYHA Class	III-ambulatory IV	III-ambulatory IV	II-III-ambulatory IV	III-ambulatory IV	II-III-ambulatory IV	II-III-ambulatory IV	III-ambulatory IV	II-III-ambulatory IV
HF hospitalization (past year)	≥ 1	≥ 1	≥ 1	≥ 1	≥ 1			≥ 1
Age (years)	> 40	> 40	> 40	≥ 18	> 18	> 40	> 18	> 17
NT-proBNP / BNP (pg/ml)	-	-NT-pro-BNP > 200 (SR) -NT-pro-BNP > 600 (AF) -BNP > 70 -BNP > 200 (AF)	-NT-pro BNP > 150 (SR) -NT-pro BNP > 450 (AF) -BNP > 50 (SR) -BNP > 150 (AF)	-NT-proBNP > 1500	-NT-proBNP > 1500 -BNP > 300	–	-NTproBNP > 125 (if LVEF > 40%)	-NT-pro BNP > 150 (SR) -NT-pro BNP > 450 (AF) -BNP > 50 (SR) -BNP > 150 (AF)
LVEF	≥ 40%	≥ 40%	≥ 40%	> 15%	Any LVEF	≥ 40%	> 15%	> 20%
Diastolic function		One or more of the following: -LA diameter > 4 cm -LAVI > 28 mL/m2 -Lateral e’ < 10 -Septal e’ < 8 -Lateral E/e’ > 10 -Septal E/e’ > 15	One or more of the following: -LA diameter > 4 cm -LAVI > 50/LAVI > 28 -Lateral e’ < 10 -Septal e’ < 8 -Lateral E/e’ > 10 -Septal E/e’ > 15	–	–	One or more of the following: -LA diameter > 4 cm -LAVI > 28 mL/m2 -Lateral e’ < 10 -Septal e’ < 8 -Lateral E/e’ > 10 -Septal E/e’ > 15	–	–
RHC	-PAWP > 15 mmHg at rest OR PAWP > 25 mmHg during exercise -RAP at rest ≤ 14 mmHg	-Exercise PAWP ≥ 25 mmHg -Exercise PAWP-RAP ≥ 5 mmHg -CI at rest ≥ 2 L/min/m^2^ -RAP at rest ≤ 14 mmHg -PVR at rest ≤ 4 WU	-Exercise PAWP ≥ 25 mmHg -Exercise PAWP-RAP ≥ 5 mm Hg -CI at rest ≥ 2 L/min/m^2^ -PVR ≤ 3.5 WU	–	–	-Exercise PAWP ≥ 25 mmHg -Exercise PAWP-RAP ≥ 5 mm Hg -CI at rest ≥ 2 L/min/m^2^ -PVR at rest ≤ 4 WU	-PAWP ≥ 15 mmHg at rest OR - PAWP ≥ 25 mmHg during exercise and RAP < 20 mmHg	-PAWP > 15 mmHg at rest OR PAWP > 25 mmHg during exercise -exercise PAWP-RAP ≥ 5 mm Hg at rest AND PAWP-RAP ≥ 10 mm Hg during exercise - CI at rest > 1.8–2.0 L/min/m^2^ (BMI < 30)
Primary outcome (efficacy)	-Number of patients with successful device implantation -% patients with reduction in PAWP at 6 months -nr. persistent left-to-right trans-device blood flow at 6 months	Change in supine exercise PAWP at 1 month (20 W, 40 W, 60 W, and 80 W) at baseline and 1 month	Hierarchical composite of: -cardiovascular death or non-fatal ischaemic stroke up to 12 months -Rate of total first plus HF events up to 24 months post-randomization -Change in KCCQ in 12 months	-Procedural success, defined as successful device implantation with no periprocedural death -Changes in NYHA functional class, quality of life, and 6-min walk distance	Composite ranking of: -All-cause death -HT or LVAD -Recurrent HHF -Recurrent worsening -Outpatient HF events and change in KCCQ overall score of at least 5 points	Change in supine exercise PAWP at peak exercise from baseline to 1 month	-	Clinical safety, device functionality, and effectiveness of the Edwards Transcatheter Atrial Shunt System
Primary oucome (safety)	-Peri-procedural and 6-month MACE (death, stroke, MI, or a systemic embolic event) or need for cardiac surgical device removal within 6 months	Cardiovascular, cerebrovascular, and renal events (MACCRE) through 1 month post-implant, including periprocedural	-Death -Non-fatal ischaemic stroke -New-onset or worsening kidney dysfunction -Major adverse cardiovascular events -Thromboembolic complications -Newly AF. Aflutter - > 30% in RV size or < 30%TAPSE at 12	Device- or procedure-related major adverse cardiovascular and neurological events	-% of patients device-related or procedure-related major adverse cardiovascular or neurological events during the first 30 days after randomization	-Composite MACE cardiac, cerebrovascular, thromboembolic events -Device/procedure-related serious adverse cardiac events	-Rate of serious adverse device-associated effects, at 3 months	Composite of major adverse cardiac, cerebrovascular, renal events, and re-intervention for study device-related complications at 30 days
Results	The IASD implant was feasible, safe, and reduced PCWP at rest and exercise	The IASD treatment group had a greater reduction in PAWP during exercise after 1 month compared with the control group	No difference in efficacy and safety outcome	V-wave implant was feasible, safe, and associated with promising efficacy data in terms of functional improvement and reduction of cardiovascular events	Ventura shunt was safe Benefit of implant in HFrEF only Harmful in HFpEF	Shunts exhibited stability with favorable safety and early efficacy signals	AFR was feasible and safe; improved symptoms and surrogate parameters of HF	APTURE Transcatheter Shunt System was safe and resulted in reduction in PAWP and improvements in HF symptoms and quality of life
Timing of functional evaluation (months)	6	1	12	12	24	6	12	3
Hemodynamic changes								
PCWP at rest (mmHg)	− 2.4	− 2.2	− 5 (legs up)	− 2	–	− 1.9	− 2.2 (HFrEF) − 5.2 (HFpEF)	–
Exercise PAWP (mmHg)	− 2.0	− 3.5	− 3.2 (20 W)	–	–	− 5.4	–	− 7.0 (20 W)
Mean RAP at rest (mmHg)	+ 2.0	+ 0.5	–	0	–	+ 1.0	0 (HFrEF) + 2.1 (HFpEF)	–
PVR at rest (WU)	− 0.2	− 0.8		–	–	+ 0.3	–	–
Echocardiographic changes								
LAVI (mL/m^2^)	1.0	− 6.3	–	− 1	+ 3.5 ml (HFrEF) + 3.8 ml (HFpEF)	− 1.6	–	–
RAVI (mL/m^2^)	+ 5.0	+ 3.0	–	–	–	+ 0.6	–	+ 1.5
TAPSE (cm)	0.0	–	–	0	+ 1 (HFrEF) 0 (HFpEF)	− 0.1	+ 0.1	–
Functional capacity changes								–
MLHFQ score	− 13	–	–	–	–	–	–	
KCCQ	–	+ 10.5	+ 10.2	73% of patients improved by > 5 points	+ 0.4 (HFrEF) − 1.7 (HFpEF)	+ 26	+ 14.9	+ 23.0
NYHA class	− 1	− 0.5	− 0.5	> + 1	–	− 1	− 1.0	–
6MWT (m)	+ 32	+ 16	–	+ 28	− 20.7 (HFrEF) − 4.3 (HFpEF)	+ 101	+ 29.6 (HFrEF) + 25.9 (HFpEF)	–

*6MWT* 6-min walking test, *AF* atrial fibrillation, *AFR* atrial flow regulator, *CI* cardiac index, *HF* heart failure, *HFpEF* heart failure with preserved ejection fraction, *HFrEF* heart failure with reduced ejection fraction, *KCCQ* Kansas City Cardiomyopathy Questionnaire, *IASD* interatrial septal device, *LA* left atrium, *LAVI* left atrial volume index, *LVAD* left ventricular assist device, *LVEF* left ventricular ejection fraction, *MLHFQ* Minnesota Living with Heart Failure Questionnaire, *NYHA* New York Heart Association, *PAWP* pulmonary artery wedge pressure, *PVR* pulmonary vascular resistance, *RAP* right atrial pressure, *RAVI* right atrial volume index, *RHC* right heart catheterization, *SR* sinus rhythm, *TAPSE* tricuspid annular plane systolic excursion

### Implant-based devices

#### Corvia medical interatrial shunt device (Corvia Medical, Tewksbury, MA, USA)

**Fig. 1:**
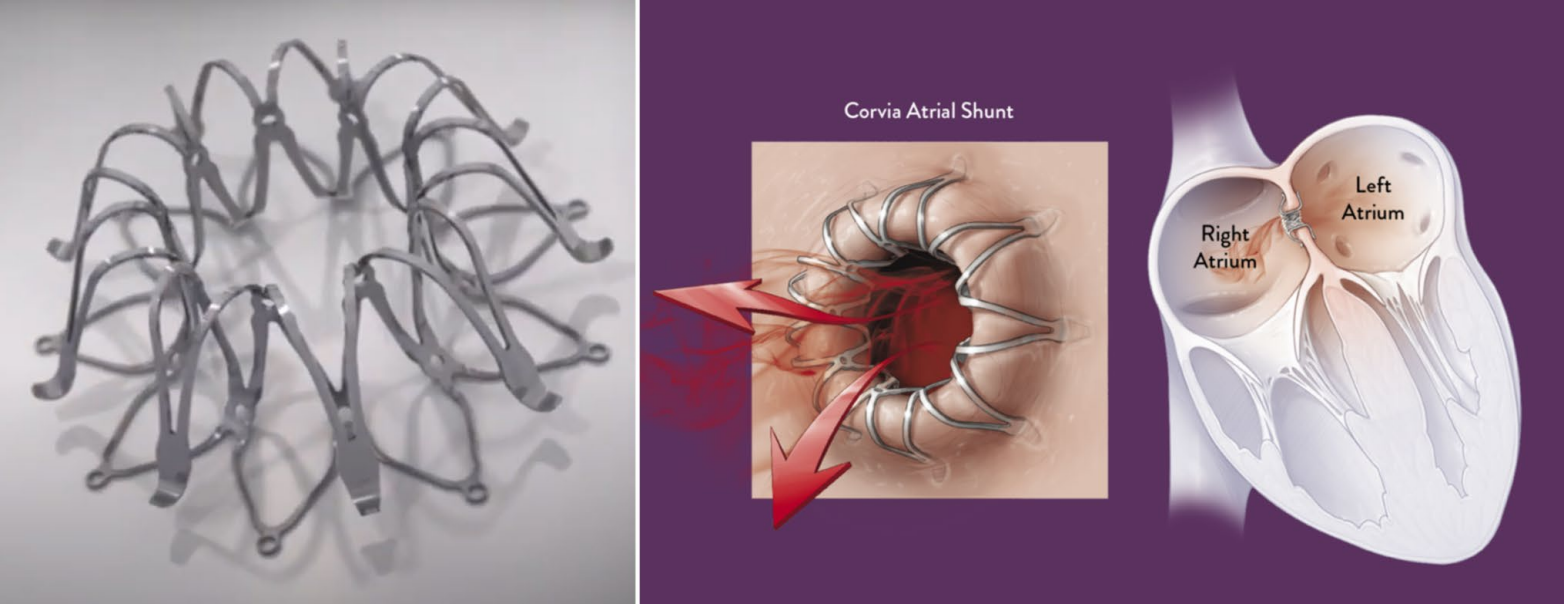
The Corvia Atrial Shunt. It consists of a nitinol frame with an 8-mm central channel, positioned within the interatrial septum. Reproduced from www.corviamedical.com

This interatrial septal device (IASD) is a self-expanding nitinol stent with two disks across the interatrial septum, featuring an 8-mm central opening (Fig. 1). It is positioned via transfemoral approach, under conscious sedation, with puncture of the interatrial septum guided by transesophageal or intracardiac echocardiography, followed by passage of the delivery system through the interatrial septum using a 16 sheath. Similar to other atrial septal devices, after the release of the LA portion, the system is retracted beyond the septum, and then, the right portion is released. Afterward, the patient receives short-term antiplatelet therapy.

This device has been used in several consecutive trials, in patients with HFpEF or HFmrEF (EF > 40%) and PAWP > 15 mmHg at rest or PAWP ≥ 25 mmHg during exercise. An initial pilot study of 11 patients demonstrated successful deployment with improvement of NYHA class and reduction in PAWP by 28% at repeat right heart catheterization performed at 30 days without major adverse cardiac events [15].

The REDUCE LAP-HF was the first multicenter, non-randomized, phase I, single-arm trial on 64 patients. At 6 months, IASD reached a 52% reduction in PAWP at rest and a 58% reduction in exercise PAWP, plus improvement in symptoms and quality of life [16]. IASD demonstrated a good safety profile (no major adverse cardiovascular events or cerebrovascular events at 6 months). At 12 months, the results were confirmed, with a modest increase in the dimensions of the right ventricle, however in the absence of right heart dysfunction [17]. This led to the REDUCE LAP-HF I a multicenter, randomized, phase II trial, on 44 patients. The inclusion criteria considered also a gradient PAWP—RA pressure > 5 mmHg. Patients were randomized to receive using a sham procedure versus IASD. IASD resulted in a reduction of the exercise PAWP at 1 month, without major adverse cardiovascular events and overall clinical benefit. At 12 months, the device remained patent in 100% of the cases [18, 19].

Because of these encouraging results, it was then conducted the REDUCE LAP-HF II, a multicentric, randomized, phase III, trial. Six hundred twenty-six patients were randomized to either the IASD or sham procedure. Unfortunately, the results showed no differences, after 1 year, between the two groups, i.e., patients treated with the IASD and the sham group. In particular, no discernible effect was seen on the composite outcome of cardiovascular death or ischemic stroke, rate of total heart failure events, or quality of life [20]. According to a post hoc analysis of the REDUCE LAP-HF II, these neutral results could be explained by the presence of non-responders (experiencing adverse events after shunt), counterbalancing the benefit observed in responders to IASD. In particular, non-responders had either a latent pulmonary vascular disease (PVD) or an implanted pacemaker [20, 21]. Latent PVD was defined by pulmonary vascular resistance (PVR) during exercise > 1.74 WU [21]. The deleterious effect of the IASD in patients with latent PVD may be explained because the flow and volume load created by the shunt on the right ventricle would (1) determine RV congestion and dysfunction, eventually minimizing or even reversing the LA to RA pressure gradient, and (2) accelerate disease progression on pulmonary hypertension. Alternatively, the hemodynamic latent PVD phenotype may underline a HFpEF profile too severe to benefit the IASD, because of “latent” RV dysfunction and/or “latent” severe tricuspid regurgitation, that might pass unrecognized at rest but that may become manifest during exercise [22]. The pathophysiological link between an implanted pacemaker and an unfavorable response to LA shunting is less clear [21]. We may speculate either a contributive role of implanted pacemaker in determining the development of tricuspid regurgitation (through lead interference), especially in a subpopulation of HFpEF at high risk of having atrial fibrillation (or bradycardia-tachycardia syndrome), which is associated with volume expansion, bi-atrial dilation, development of tricuspid regurgitation, and afterload-independent right ventricular failure [23].

Supporting the previously identified responder group hypothesis and mechanism, in another post hoc analysis of the REDUCE LAP-HF II trial, over 2 years of follow-up, atrial shunting led to more favorable changes in cardiac structure/function in responders compared with non-responders. Responders (vs. non-responders) randomized to the shunt had smaller increases in RV EDV RV end-systolic volume, RV/LV ratio, and RVEF [24].

This led to a still ongoing dedicated trial, the RESPONDER HF (Reevaluation of Atrial Shunt Device in a Precision Medicine Trial to Determine Efficacy in Mildly Reduced or Preserved Ejection Fraction Heart Failure), a randomized, double-blinded trial which tested the safety and efficacy of IASD in patients with symptomatic HF, EF > 40%, and absence of latent pulmonary vascular disease and implanted pacemaker.

#### Ventura V-WAVE interatrial shunt (V-Wave, Caesarea, Israel)

**Fig. 2:**
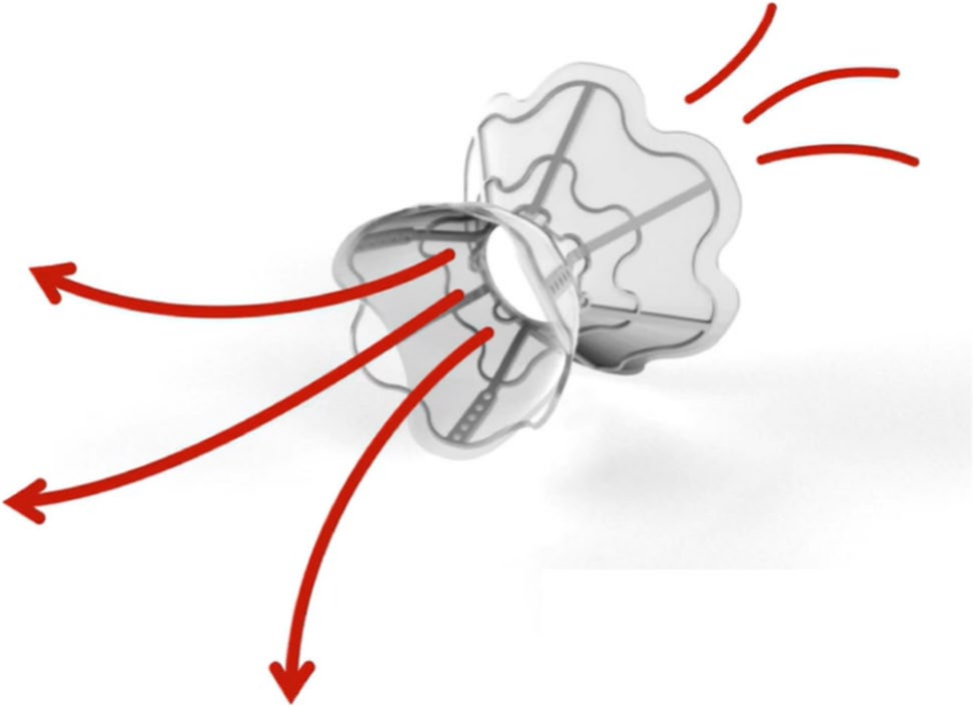
The V-Wave Ventura Interatrial Shunt System. It is a hourglass-shaped implantable device in the interatrial septum. Reproduced from https://vwavemedical.com/

This device is conceptually similar to the previous one. The main differences are the hourglass shape, the polytetrafluoroethylene skirt over a nitinol mesh, and a smaller (5.1 mm) central lumen (Fig. 2). The first-generation device had a porcine pericardium tissue valve to allow a unidirectional left-to-right flow; the valve was then removed because of the pannus formation resulting in shunt stenosis or occlusion in 50% of patients in a dedicated trial [25].

One of the most important differences from the IASD is also the target population in which the device has been tested: both patients with HFpEF and HFrEF. The first, single-arm, pilot studies proved the feasibility and the safety of the procedure [25, 26] and showed improvement in NYHA class, quality of life, and 6-min walking distance, as well as a reduction in PAWP at 3 and 12 months after shunting, comparing with baseline. In detail, at 3 months, 78% of patients improved from NYHA functional class III or IV at enrollment to class I or II, and at 12 months, 60% continued to improve. The 6-min walk distance increased by 41 m at 3 months and by 28 m at 12 months. Patients with patent shunts (at 12-month follow-up, 14% of patients have total occlusions with this first-generation device) exhibited significant improvements in PCWP (from 23.3 ± 5.4 mmHg at baseline to 18.0 ± 4.0 mmHg at 12 months), without worsening of right atrial or pulmonary artery pressures [25].

The RELIEVE-HF trial (Reducing Lung Congestion Symptoms in Advanced Heart Failure) was a prospective, multicenter, randomized trial recruiting 508 HFrEF and HFpEF patients, with the exclusion of patients with severe pulmonary hypertension and right ventricular dysfunction [27]. The eligibility criteria were ischaemic or non-ischaemic cardiomyopathy with either reduced or preserved LV EF and documented HD for at least 6 months, NYHA class II, III or ambulatory IV, HF hospitalization during prior 12 months OR BMI-adjusted NT-proBNP levels > 1500 pg/ml or BNP > 300 pg/ml, receiving optimized GDMT for drugs and devices, 6 mWT between 100 and 450 m. Some of the exclusion criteria were age < 18 years, BMI > 45 or < 18 kg/m2, RV dysfunction, untreated severe valvular or coronary heart disease, intracardiac thrombus, etc. The primary safety endpoint was one of the major cardiovascular and neurological events at 30 days; the primary efficacy endpoint was a composite of death, transplantation, LV assist device implantation, recurrent HF hospitalization, and change in quality of life. Notably, differently from other trials in the field, invasive hemodynamics was not a criterion to evaluate candidacy for the procedure.

The study has been recently published [27] and showed that transcatheter implantation of the V-wave interatrial shunt was positive on the safety endpoint but was neutral for the primary effectiveness endpoint, because it did not reduce symptoms or improve heart failure patient’s prognosis. Results from a pre-specified stratified analysis suggested that interatrial shunt implantation may be beneficial in patients with HFrEF. Indeed, in HFrEF, it did not meet the primary effectiveness endpoint (a hierarchical composite of all-cause death, cardiac transplantation, implantation of LV assist device, HF hospitalization, outpatient HF worsening, quality of life improvement), but it met the secondary composite event endpoints and secondary effectiveness endpoints, including all-cause death, cardiovascular death, and heart failure hospitalization. The annualized rate of events was 49% in the shunt group and 88.6% in the placebo group (relative rate ratio 0.55, *p* < 0.0001). Instead, at the same pre-specified analysis restricted to HFpEF patients, the Ventura device resulted in harm, with negative results in the primary effectiveness endpoint and in the secondary composite event endpoints. In patients with HFpEF, the annualized rate of events was 60.2% in the shunt group and 35.9% in the placebo group (relative rate ratio 1.68, *p* < 0.0001). Notably, inclusion and exclusion criteria for this device differed from those adopted with other devices, being more inclusive and enrolling more advanced HFpEF patients (i.e. those excluded in trials with other shunting devices).

#### Occlutech atrial flow regulator (Occlutech AG, Switzerland)

**Fig. 3:**
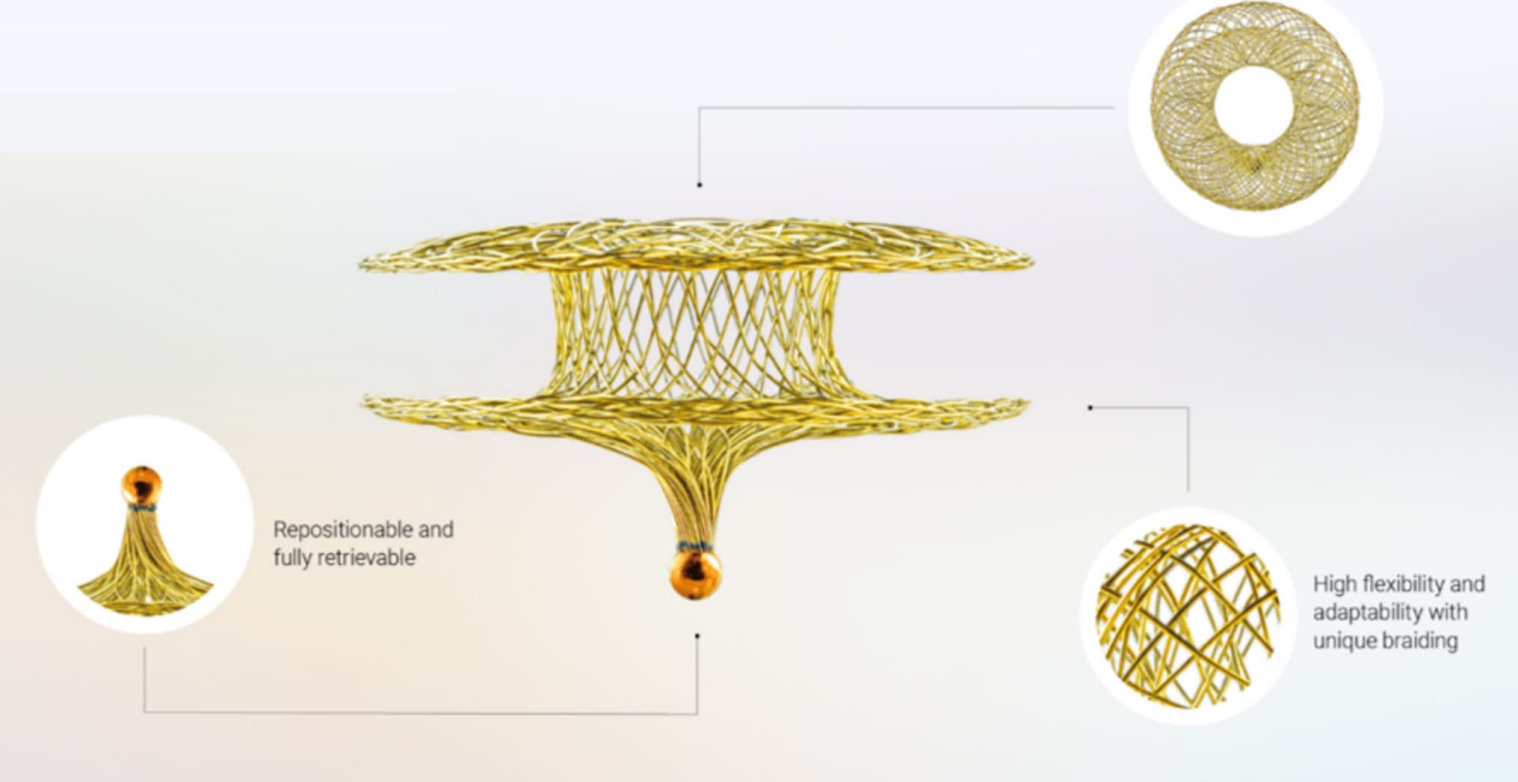
The Occlutech Atrial Flow Regulator**.** It has a two-disk configuration with a central fenestration to be implanted in the interatrial septum. The device is available with two different fenestration diameters, according to the desired size of interatrial communication using a sizing algorithm. Reproduced from https://occlutech.com/afr/

Occlutech atrial flow regulator is a double-disk device with self-expanding nitinol wire, available in different sizes (4, 6, 8, 10 mm) according to the level of PAWP and the thickness of the septum (Fig. 3). Like the V-wave, it has been employed in patients with both HFpEF and HFrEF in a dedicated trial, the AFR-PRELIEVE [28], improving NYHA class and reducing PAWP.

#### Apture shunt (Edwards Lifesciences, Irvine, California)

**Fig. 4:**
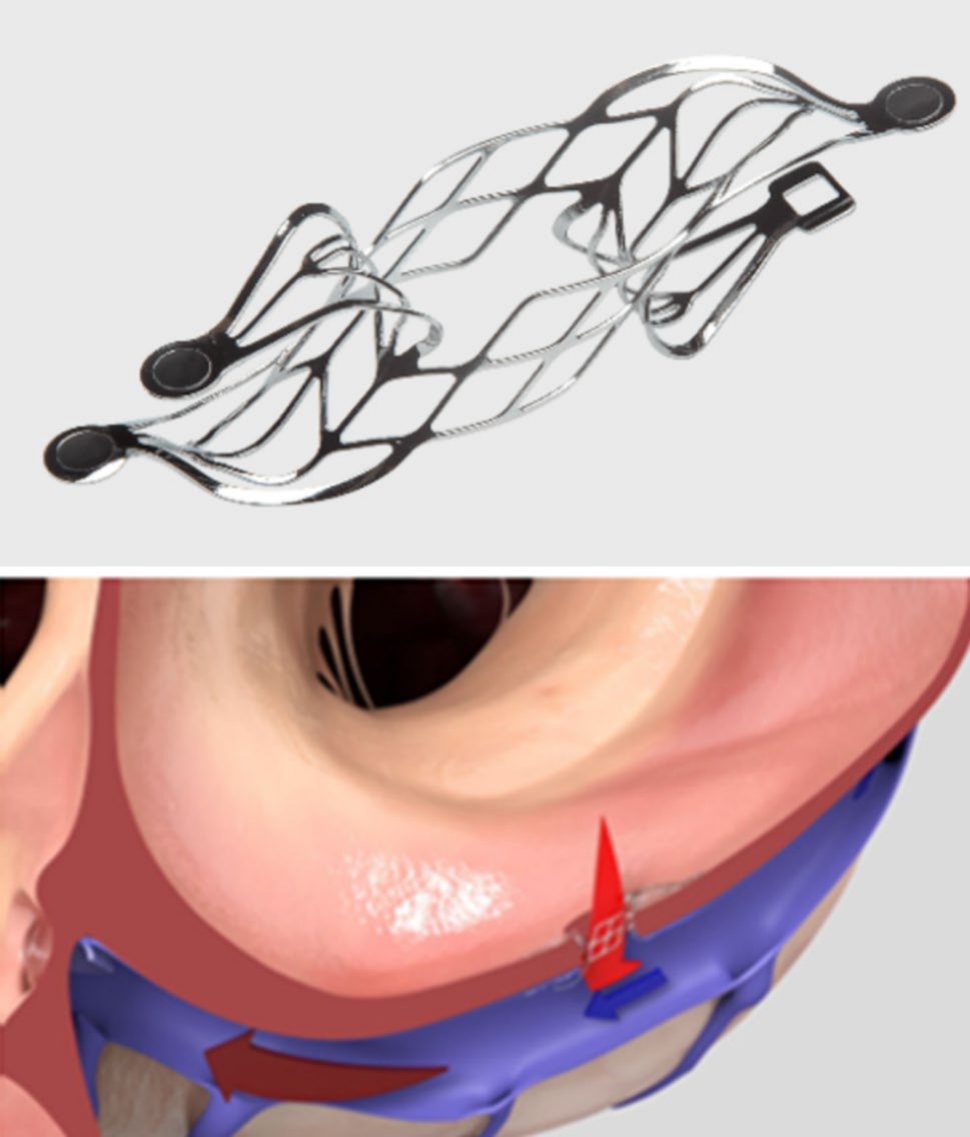
The Apture Trans-catheter Shunt System. It establishes a left atrium to coronary sinus shunting following percutaneous atriotomy from the coronary sinus and positioning a nitinol-based shunt. It allows flow from the left atrium to the coronary sinus and subsequently to the right atrium. Reproduced from https://www.edwards.com/healthcare-professionals/trial/altflow

The transcatheter atrial shunt system is a nitinol mesh, which creates a 7-mm shunt between the LA and the coronary sinus (Fig. 4). Its efficacy on quality of life and exercise PAWP has been proven in a preliminary pilot study on 11 patients and in a single-arm study on 87 patients [29, 30]. Since it completely spares the interatrial septum, it has theoretical advantages, including a lower likelihood of paradoxical embolization and preservation or RA dynamics. However, these theoretical advantages are counterbalanced by a more complex implantation technique, with high but suboptimal feasibility (90%) [28], and a signal for postimplant adverse events (2 emergent cardiac surgery at 30 days in 78 implanted patients) [30], higher with what generally reported in the experiences conducted so far with other devices.

### Implant-free devices

Since the implant-based devices present a relatively large outer diameter, which might limit possible future transseptal percutaneous procedures and impact on atrial dynamics, systems to generate a shunt without leaving devices on the interatrial septum have been successively developed.

**Fig. 5:**
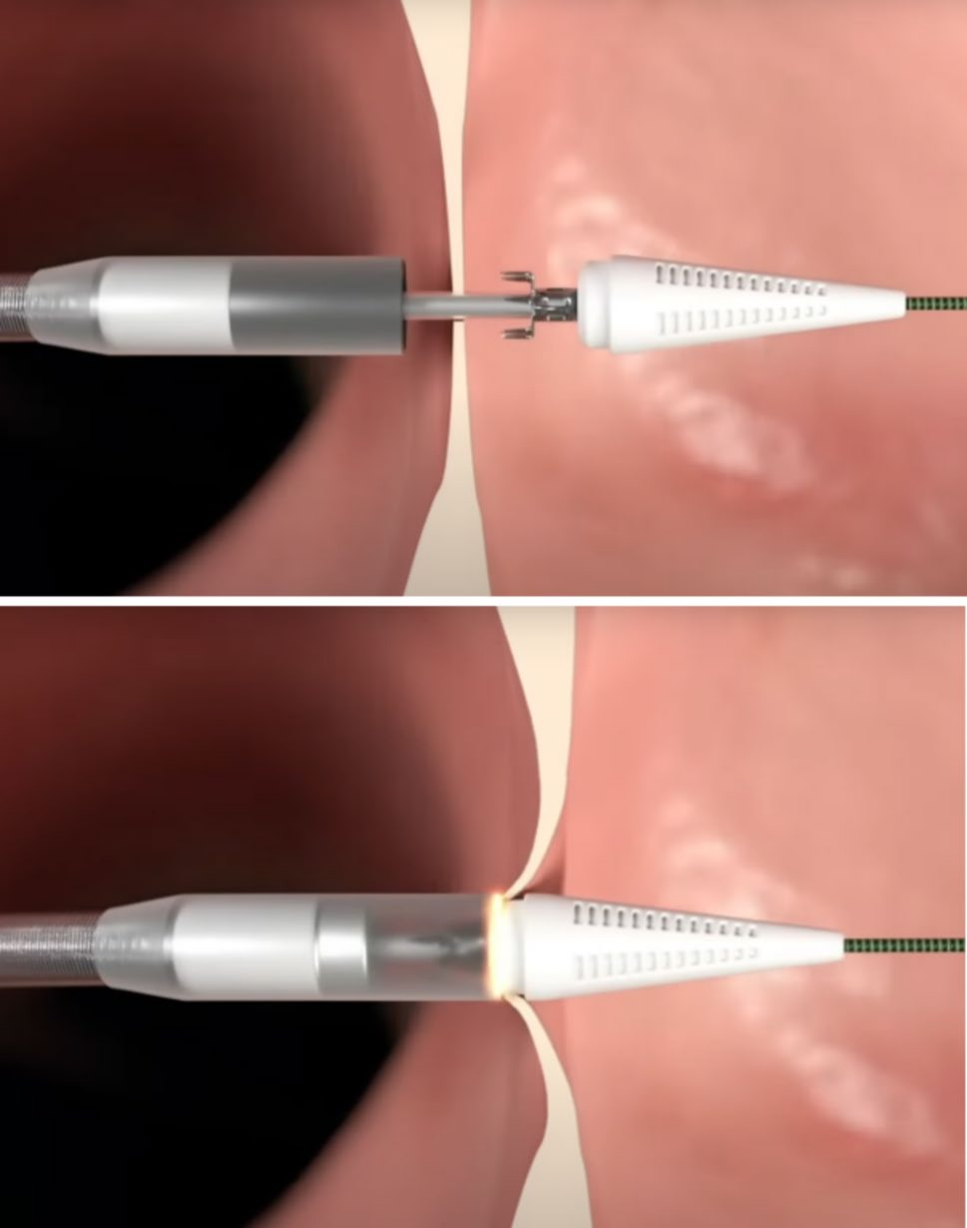
The Alleviant System. It is a no-implant-based device. It uses radiofrequency energy to securely capture, excise, and extract a precise disk of tissue from the interatrial septum. Reproduced from https://www.alleviantmedical.com/alleviant-system

- *Alleviant system*. This system consists of a specifically designed catheter that is advanced through the fossa ovalis, grasps the interatrial septum, and can perform a radiofrequency excision of the interatrial tissue, creating a shunt of 6–7 mm diameter (Fig. 5). This radiofrequency-based excision assures long-term patency of the shunt. This system has been mainly studied in patients with HFpEF, demonstrating improvement in quality of life at 6 months and reduction of PAWP [31, 32]. A large, multicenter, randomized, sham-controlled trial, the ALLAY-HF (Safety and Efficacy of the Alleviant System for No-Implant Interatrial Shunt Creation in Patients With Chronic Heart Failure) is currently underway and seeks to enroll 400 to 700 patients with HF and EF ≥ 40%
- *InterShunt device*. This system consists of a percutaneous device that can excise a 6-mm circular section of interatrial septum tissue. A pilot study on 10 patients demonstrated the safety and patency of the shunt at 90 days [33].
- *NOYA Radiofrequency Interatrial Shunt System*. It employs a radiofrequency ablation catheter, with the smallest diameter size of 4 mm, up to 10 mm. The first study (RAISE Trial, 2022) on 10 patients with EF > 40% demonstrated patency at 6 months on 7 patients and improvement in symptoms and BNP [34]. A prospective, multicenter trial (RAISE TrialII) is still ongoing.

## When: persisting uncertainties

The therapeutic window with optimal risk–benefit ratio of interatrial shunt devices in HFpEF and HFmrEF has not been definitely ascertained, despite they constitute the largest HF population studied up to now. Signals coming from large, sham-controlled clinical trials (REDUCE LAP-HF II [20] and RELIEVE-HF) seem to confirm the need for proper clinical and hemodynamic phenotyping of patients to detect good candidates [35, 36] and suggest that patients with even subclinical right heart involvement may not benefit of this procedure. Indeed, the shunt may only be effective in the presence of a flow-guiding pressure difference between the LA and the RA (with LA pressure > RA pressure) [14] and in the absence of overt or latent pulmonary vascular disease/right ventricular dysfunction [21, 22]. Only ongoing trials may help answer this question. Additionally, follow-up data from past and ongoing trials will help capture the patterns of right heart remodeling after the creation of the interatrial shunt. Despite the observation of right heart enlargement at a 2-year follow-up in the REDUCE LAP-HF II trial, this seemed to be without adverse consequences and, most importantly, not associated with new-onset right ventricular dysfunction [24]. In addition, it is important to recognize the need for standardized evaluation of atrial mechanics in the setting of atrial shunt therapy. Investigators of the REDUCE LAP-HF-TRIAL noticed that implantation of IASD resulted in heterogenous changes in the LA volume. A favorable decrease in LA volume could be present in patients with higher LA compliance and right atrial reservoir strain [37].

No-implant devices have a theoretical advantage over implant-based devices. First, avoiding foreign material on the interatrial septum may interfere with atrial dynamics and may trigger atrial fibrillation in the short term [38, 39]. Indeed, LA dynamics may be already impaired in HFpEF patients, and HFpEF and atrial fibrillation may represent two sides of the same coin [40]. Nonetheless, signals coming from the REDUCE LAP-HF II trial seem to be reassuring on these sides [41], suggesting that the favorable effects of the shunt may counterbalance its potential negative effects. Second, albeit still not reported, device-related thrombosis and infection exist as a remote possibility [42].

## Conclusion

The creation of a LA shunt in HF patients represents a promising therapeutic strategy formulated from the theoretical assumption of reducing the high left heart filling pressures, in the attempt to alleviate patients’ breathlessness and improve quality of life and hopefully prognosis. Different types of devices have been tested. However, the latest data have raised questions on the optimal selection of HF(pEF) candidates to LA shunting devices. Ongoing trials may help define the patients’ phenotype across HF heterogeneity that could effectively benefit from LA shunting devices.
