# Correction to: Left atrial shunting devices: why, what, how, and… when?

**DOI:** 10.1007/s10741-026-10642-2

**Published:** 2026-06-11

**Authors:** Leila Anna De Lorenzo, Claudia Baratto, Davide Sala, Giovanni Battista Perego, Sergio Caravita

**Affiliations:** 1 Università Vita E Salute San Raffaele, Milan, Italy; 2 Department of Cardiology, San Luca Hospital, IRCCS Istituto Auxologico Italiano, Milan, Italy; 3 Department of Management, Information and Production Engineering, University of Bergamo, Edificio C, Via Pasubio, 24044 Dalmine, Bergamo Italy; 4 Dyspnea and Pulmonary Hypertension Center, Ospedale San Luca, Piazzale Brescia 20, 20149 Milano, Italy

**Correction to: Heart Failure Reviews (2025) 30:685–696**

10.1007/s10741-025-10485-3

In the original version of this article, the author affiliations were incorrectly presented. The fourth affiliation, “4 Dyspnea and Pulmonary Hypertension Center, Ospedale San Luca, Piazzale Brescia 20, 20149 Milano, Italy” was included in error and should be removed.

The corrected affiliations list for Sergio Caravita should read:

2. Department of Cardiology, San Luca Hospital, IRCCS Istituto Auxologico Italiano, Milan, Italy.
3. Department of Management, Information and Production Engineering, University of Bergamo, Edificio C, Via Pasubio, 24044 Dalmine, Bergamo, Italy.

The authors apologize for any inconvenience this error may have caused.

